# Can We Predict the Efficacy of Anti-TNF-α Agents?

**DOI:** 10.3390/ijms18091973

**Published:** 2017-09-14

**Authors:** Loris Riccardo Lopetuso, Viviana Gerardi, Valerio Papa, Franco Scaldaferri, Gian Lodovico Rapaccini, Antonio Gasbarrini, Alfredo Papa

**Affiliations:** 1 Internal Medicine and Gastroenterology Department, Fondazione Policlinico Gemelli, Catholic University of Rome, 00168 Rome, Italy; lopetusoloris@libero.it (L.R.L.); vivianagerardi@libero.it (V.G.); francoscaldaferri@gmail.com (F.S.); gianludovico.rapaccini@policlinicogemelli.it (G.L.R.); antonio.gasbarrini@unicatt.it (A.G.); 2 Digestive Surgery Department, Fondazione Policlinico Gemelli, Catholic University of Rome, 00168 Rome, Italy; valerio.papa@unicatt.it

**Keywords:** inflammatory bowel disease, Crohn’s disease, ulcerative colitis, biologics, anti-TNF-α, predictors, short-term efficacy, long-term efficacy

## Abstract

The use of biologic agents, particularly anti-tumor necrosis factor (TNF)-α, has revolutionized the treatment of inflammatory bowel diseases (IBD), modifying their natural history. Several data on the efficacy of these agents in inducing and maintaining clinical remission have been accumulated over the past two decades: their use avoid the need for steroids therapy, promote mucosal healing, reduce hospitalizations and surgeries and therefore dramatically improve the quality of life of IBD patients. However, primary non-response to these agents or loss of response over time mainly due to immunogenicity or treatment-related side-effects are a frequent concern in IBD patients. Thus, the identification of predicting factors of efficacy is crucial to allow clinicians to efficiently use these therapies, avoiding them when they are ineffective and eventually shifting towards alternative biological therapies with the end goal of optimizing the cost-effectiveness ratio. In this review, we aim to identify the predictive factors of short- and long-term benefits of anti-TNF-α therapy in IBD patients. In particular, multiple patient-, disease- and treatment-related factors have been evaluated.

## 1. Introduction

Inflammatory bowel disease (IBD), including Crohn’s disease (CD) and ulcerative colitis (UC), are chronic disorders that predominantly affect the bowel as a result of an abnormal immune response to enteric microbes in genetically-predisposed individuals [1]. The progressive organ damage results in a considerable disability for patients and, in turn, an economic and social burden [2,3].

The use of biological therapies, in particular anti-tumor necrosis factor (TNF)-α agents, has spread increasingly in the last two decades thanks to their potential and unique ability of altering the natural history of IBD. In fact, both controlled trials and “real life” studies showed that anti-TNF-α agents, including infliximab (IFX), adalimumab (ADA), golimumab (GOL) and certolizumab pegol (CER), are able to induce and maintain clinical remission and to reduce the need for surgery and hospitalization. Furthermore, they lead to an improvement in quality of life and also to a reduction in direct and indirect costs associated with these chronic debilitating disorders [4].

Unfortunately, anti-TNF-α agents are not a magic bullet for all IBD cases. Firstly, side-effects can be severe and life-threatening in some patients, mainly due to infective events and then to immunogenicity, with the formation of antibodies-to-anti-TNF-α and a consequent loss of response (LOR) to these drugs over time. Literature data report that primary non-response to anti-TNF-α induction therapy (assessed not before weeks 8–12 after starting treatment) occurs between 20–40% of patients in clinical trials and in 10–20% in “real life” series. Secondary LOR is also a common clinical problem with incidence ranging between 23 and 46% at 12 months after anti-TNF-α initiation [5]. Thus, the availability of predictors of efficacy for anti-TNF-α treatment would be extremely useful in clinical practice in order to optimize treatments and to minimize side-effects and costs. This is particularly true given the increasing availability of biological therapies against other specific targets (i.e., anti-α4β7 integrin, anti-p40 subunit of interleukin-12 and interleukin-23).

Therefore, the aim of this review was to explore any possible predictors of efficacy for the anti-TNF-α agents approved for IBD, using available data from clinical trials and “real life” studies.

## 2. Definition of Biological Treatment Effectiveness

The first difficulty in recognizing the predictors of response to treatment with anti-TNF is the lack of a unique definition of effectiveness that often varies according to the different studies. In addition, the definition of efficacy has been subjected to a continuous evolution in recent years. Indeed, clinical remission assessment was generally based on the use of several clinical scores. However, all of them have a share of subjectivity that does not make them sufficient to fully evaluate the effectiveness of a pharmacological treatment, so other parameters have been evaluated in order to better define remission in IBD patients in a more objective way. For example, the criterions of steroid-free clinical remission or of mucosal healing have been added, giving rise also to the concept of “deep remission”. Lastly, a “composite remission measure” has been coined with clinical and endoscopic outcomes associated to biological markers, such as C-reactive protein (CRP) or faecal calprotectin [6,7]. Consequently, short- and long-term outcomes of an anti-TNF-α treatment could substantially differ depending on the study, rendering data interpretation even more complex. For these reasons, the different definitions of treatment outcome have been considered for the purposes of this review (see Table 1).

## 3. Predictors of Anti-Tumor Necrosis Factor (TNF)-α Efficacy

We can schematically distinguish the predicting elements of efficacy for anti-TNF-α agents in IBD patients in three groups: (1) patient-related factors; (2) disease-related factors (i.e., the specific features of the disease); (3) treatment-related factors.

The first two groups include independent aspects that exist before the initiation of anti-TNF-α treatment, while the third group includes factors usually assessed in the short- (i.e., after induction period, within 12 weeks) and/or in the long-term (i.e., after one year of treatment) after the start of anti-TNF-α therapy.

### 3.1. Patient-Related Factors

Several patient-related factors have been associated with the efficacy of anti-TNF-α treatments in IBD. A number of epidemiological predictors of response have been reported. However, they appear of limited clinical utility for most patients and studies in this field have often produced controversial results.

#### 3.1.1. Gender

The relationship between gender and IBD risk is still unclear. Also, results obtained from studies in experimental models are controversial [8]. On the other side, a retrospective study on a cohort of UC patients showed the absence of gender influence on the response to IFX [9]. Conversely, among 125 patients with steroid-refractory UC evaluated retrospectively and treated with IFX, female gender was correlated with an increased remission rate at 1 year and cumulative non-colectomy rate [10].

#### 3.1.2. Age

A positive correlation between younger age and response to anti TNF agents was found in a study on 100 UC patients conducted by Ferrante et al. while opposite results came from a study conducted by Jakobovits et al. on 30 refractory UC patients, where younger age correlated to an increased risk of surgery, although the power of this study was limited by the limited number of patients [11]. In a retrospective study, age was not significantly associated to response to IFX in a UC population [9]. Of note, a larger retrospective and prospective multicenter study compared 250 CD long-term users of IFX (>5 years of treatment) with 290 CD non-long term users to identify prognostic factors. The latter included primary non-responders to IFX, patients who experienced a loss of response and those who faced an adverse event that needed the discontinuation of treatment. Earlier age at the start of IFX (mean age 33.1 ± 13 vs. 38.2 ± 14) was associated with long term and thus beneficial use [12].

#### 3.1.3. Smoking

Smoking did not affect the response rate to IFX in UC patients analyzed retrospectively by Gonzalez-Lama et al. [9], as well as in the report by Ferrante et al., where colectomy rate was not increased in the subgroup of 9 active smokers with UC [13]. On the other hand, smoking was identified as a risk factor for worsening CD course [14] and, accordingly, non-smoking was associated with higher rates of response and longer duration of response to IFX in a population of 100 CD patients [15]. Similar results were obtained in a prospective study that evaluated 74 IFX-treated CD patients. Univariate analysis and multiple logistic regression confirmed smoking as an independent marker of response with an Odds Ratio of 0.22 and 0.24, respectively. Furthermore, one-year relapse assessment showed significant diverse rates between smokers and non-smokers (100% vs. 39.6% with a relative risk of 3.2) [16].

Conversely, further analyses produced opposite results. Indeed, smoking habits were not retained as predictors for response in a cohort of 240 CD IFX-treated patients with refractory luminal CD or fistulizing CD assessed at 4 or 10 weeks after the first infusion, respectively [17]. Correspondingly, among 200 CD IFX-treated patients evaluated for at least 6 months of follow up, nonsmokers and smokers had similar response rates and similar durations of response regardless of disease phenotype (e.g., luminal or fistulous disease) [18]. Similar results were observed in an Italian cohort of 573 CD patients with luminal refractory or fistulizing disease or both treated with Infliximab [19].

#### 3.1.4. Body Mass Index (BMI)

Increased BMI and obesity represents a new growing issue in the management of IBD patients and have been indicated to condition response to anti-TNF-α. In a randomized, placebo-controlled trial including patients with moderately to severely active UC treated with ADA, clinical remission rates after 8 weeks resulted to be more than twice in patients lower than 82 kg vs. patients above 82 kg in the 160/80 mg dose group (24.2% vs. 9.6%) [20]. On the other side, in a retrospective study that evaluated 124 IBD patients, naive to anti-TNF-α, increased body weight represented a prognostic factor for earlier loss of response to IFX in both CD and UC. In particular, BMI > 30 kg/m was correlated with an increased risk of flares in CD patients (adjusted hazard ratio (HR): 3.03). Similar results were observed in overweight (BMI > 25 kg/m) UC patients (HR: 9.68). Earlier IBD flare was correlated to increased BMI and mass (considered as continuous variables) in both CD and UC (adjusted HR: 1.06 per unit increase in BMI and 1.02 per kg increase in body mass; adjusted HR: 1.3 per unit increase in BMI and 1.11 per kg increase in body mass, respectively) [21]. Also, in a retrospective study on 120 pediatric CD and UC patients, response to anti-TNF-α (both IFX and ADA) was associated with lower weight and BMI [22]. Conversely, in a CD pediatric population treated with IFX, loss of response was associated with low BMI at diagnosis and lower height Z scores prior to induction [23].

### 3.2. Disease-Related Factors

Disease-associated features represent a crucial prognostic factor on the subsequent efficacy of anti-TNF-α agents. They include not only clinical factors, such as clinical behavior, age at diagnosis, disease-duration, previous or concomitant medications, previous intestinal resections (for CD patients), but also laboratory factors such as CRP levels and genetic factors or gene expression signature assessed prior to start anti-TNF-α agent.

#### 3.2.1. Age at Diagnosis

In a retrospective, single-center study on 100 CD patients by Papamichael et al., age at diagnosis of 25 years or older was an independent predictive factor for sustained clinical remission after IFX discontinuation while in clinical remission. Sustained clinical remission was defined as maintenance of remission without the necessity of medical therapy or surgery during the follow-up period (median 9.7 years) [24]. However, this study was based only on physician global assessment and not on clinically validated scoring systems, and endoscopic data at the time of IFX cessation were available only for few patients. Another retrospective study on 51 IBD patients (17 CD, 30 UC and 4 IBD unclassified) in deep remission at the time of cessation of anti-TNF-α agent did not find age as a predictor of relapse after a median of 36 months [25]. Age at diagnosis was not a predictive factor also in the abovementioned study on CD long-term users of IFX (>5 years of treatment) compared with CD non-long term users [12].

#### 3.2.2. Disease Duration

Disease duration has been proposed following the idea that shorter disease duration could reflect a better response to early treatment. This seems to be not true in UC. Indeed, disease duration before starting IFX in UC patients was not correlated to response to IFX according to two different series from Oxford and from Spain. The first study reviewed the rate of colectomy after IFX for UC in a retrospective cohort study of 30 active UC patients treated between 2000 and 2006 (Oxford cohort) [26]. The latter evaluated clinical response, steroid-free remission and colectomy rates in a multicenter retrospective survey of 47 UC patients treated with IFX within a mean follow up of 4.7 months [9]. Moreover, the analysis of the Active ulcerative Colitis Trials (ACT) 1 and 2 added new data regarding prognostic factors for colectomy in patients with moderate to severe UC treated with IFX. Among 630 patients, duration of colitis ≤3 years strongly augmented the risk for colectomy (HR: 0.36, *p* < 0.001) along a follow-up period of 54 weeks [27].

In CD patients, post-hoc analyses from large clinical trials underlined that a duration of disease <2 years was correlated to an increased rate of response to CER or ADA than those with more long-standing disease [28,29]. This concept was also shown in CD pediatric populations treated with IFX [30]. In this scenario, the “Randomized, multicenter, open-label study to Evaluate the safety and efficacy of Anti-TNF-α Chimeric monoclonal antibody in pediatric subjects with moderate to severe Crohn’s disease (REACH study)” evaluated the safety and efficacy of IFX in children with moderate to severe active Crohn’s disease [31]. The significantly higher response rates in this trial compared to those found in adults could directly suggest a possible connection to earlier intervention or younger age of treatment [17]. Furthermore, Papamichael et al. found in their abovementioned retrospective study that disease duration <1 year at the time of IFX discontinuation correlated with a sustained clinical remission [24]. Conversely, no relationship was observed in other studies on disease duration [15,16,18]. Overall, although an earlier approach with anti-TNF-α agents when inflammatory phenotype prevails is intriguing, the correlation between shorter duration of disease and a more favorable effect of anti-TNF-α is still controversial.

#### 3.2.3. Disease Severity

Multiple studies have evaluated the impact of disease severity on the efficacy of anti-TNF-α administration both in UC and CD. No statistically significant correlation between disease activity and rate of colectomy was found in a small cohort of 30 UC patients treated with IFX [26]. Similarly, no correlation was observed between UC severity and early response to IFX in a single center study conducted on 100 UC patients [32]. In another study on 89 patients with moderate to severe UC treated with IFX, disease severity was a predictor of primary non-response [33]. In the ACT 1 and 2 trials, a baseline Mayo score ≥10 before starting IFX significantly increased the colectomy risk (HR: 1.84, *p* = 0.01) [27].

On the other side, in another cohort of 90 UC out-patients treated with IFX and followed for 14 weeks, a positive correlation between UC activity (evaluated by Colitis Activity Index) and response was noted. However, only a few severe cases were enrolled [34].

Overall, patients with increased disease severity seem to be less prone to anti-TNF-α treatment response. This has also been confirmed in a placebo-controlled trial of patients with refractory UC disease naïve to biologics and treated with ADA. Short-term efficacy evaluation showed that an augmented disease severity at baseline negatively correlated with remission rates at week 8 [20]. In particular, a Mayo score ≥10, a CRP ≥10 mg/L and an extensive disease seemed to be negative prognostic factors [20]. Nonetheless, these elements had not a significant impact on the overall remission rates. Furthermore, in a prospective study on 21 patients with moderate-to-severe UC treated with GOL, non-responders had a worse disease severity at baseline (defined by endoscopic Mayo score) when compared with responders (*p* = 0.048) [35].

In CD, the lack of a clear agreement on disease severity definition is more evident with a consequent less defined scenario. In a retrospective study on 425 CD patients, stricturing and penetrating disease at the time of anti-TNF-α prescription, two indicators of increased disease severity, were significantly associated with the risk for surgery (adjusted HR: 6.17; 95% CI: 2.81–13.54 and adjusted HR: 3.39; 95% CI: 1.45–7.92, respectively) [36]. In a randomized control trial on 89 patients with active CD and treated with CER, reduced CD severity (assessed with Crohn’s Disease Activity Index (CDAI)) was associated with higher response rates. This coincided also with increased drug concentration at week 6 vs. patients with reduced drug levels [37]. This is in line with the hypothesis that a reduced efficacy in more severe disease could be due to an increased clearance of the drug into the stool through the ulcerated mucosa both in CD [38] and UC [39], as also suggested by animal colitis models [40].

#### 3.2.4. Disease Extent

Disease extent could also affect response to anti-TNF-α treatment. Among different factors explored, extent of the disease was the only predictive factor related to higher response rates to IFX in an open-label study with a mean follow-up period of 8 months on 47 UC patients. In this trial, pancolitis correlated with higher response rates compared with left sided colitis (*p* = 0.02) [9]. On the other side, extensive colitis was negatively related to response in the short term in a multicenter randomized controlled trial on moderately to severely active ulcerative colitis patients treated with ADA [20]. Finally, no correlation was found in the abovementioned Oxford cohort treated with IFX [26].

Isolated Crohn’s colitis seems to be related to a better response to IFX [16,17]. Furthermore, localized ileal stricturing disease could be associated with primary nonresponse to anti-TNF-α treatment [38]. However, one study underlined that isolated ileal stricturing disease treated with IFX had the same risk of surgery of stricturing disease of other unspecified sites [36].

#### 3.2.5. Disease Phenotype

Disease phenotype as defined by the Montreal classification [41] can represent a useful factor in predicting the response to anti-TNF-α treatment. In general, patients with a pure inflammatory disease-behavior should be expected to have an increased benefit from anti-TNF-α therapy than patients with a complicating phenotype. In fact, in a study on 201 CD patients treated with ADA, only luminal disease was positively correlated with both primary response and sustained clinical remission at week 52 (OR: 3.89; 95% CI: 1.43–10.6; *p* = 0.008). Conversely, patients with luminal and fistulizing disease had lower remission rates at each time point than those with a luminal phenotype alone (week 12: 42.5% vs. 56.3%, *p* = 0.06) [42].

Similarly, fistulizing CD disease has been demonstrated to have a lower short-term response rate on a cohort of 240 CD patients treated with IFX [17]. At the same time, fibrostenotic disease can have a lower primary response to IFX as shown in a retrospective cohort study of 425 CD patients. In these cases, surgical resection or endoscopic dilation therapy have been shown to be more efficacious [36]. However, other authors did not describe a clear association between phenotype and response [15,19].

#### 3.2.6. Previous Intestinal Resections (for Crohn’s Disease (CD) Patients)

Past history of surgical resection may influence the response to anti-TNF-α in CD patients. Intestinal resection before starting an anti-TNF-α was correlated with a reduced response rate in a cohort of 240 CD patients treated with IFX (OR: 0.429, 95% CI: 0.233–0.787, *p* = 0.006) [17]. Similarly, previous surgery predicted a worse response for fistulizing disease in a multicentric Italian open study on 573 patients treated with IFX and characterized by luminal refractory CD or fistulizing disease or both of them (OR: 0.53, 95% CI: 0.30–0.93) [19]. On the contrary, no association between response evaluated after 4 weeks and previous surgery was found in a prospective study on 74 IFX-treated patients with CD.

#### 3.2.7. Laboratory (Biological) Factors

Many biological variables have been also considered as possible predicting factors of response to anti-TNF-α. Among these, CRP levels represent probably the most studied factor, which seems to have a more important influence in CD compared with UC.

Several studies evidenced a correlation between elevated CRP levels and colectomy in UC patients treated with anti-TNF-α [11]. In a study on 121 UC patients treated with IFX and followed for a median of 33 months, a pre-treatment CRP value ≥5 mg/L was an independent predictor for colectomy (HR: 14.5, *p* = 0.006) [13]. In the ACT trials, a pre-infusion CRP level ≥2 mg/L was associated with an increased colectomy rate (HR: 1.73, *p* = 0.04) [27]. High CRP (≥10 mg/L) at baseline were associated with reduced remission rates at week 8 in the placebo-controlled trial of patients with refractory UC disease naïve to biologics and treated with ADA [20]. On the contrary, a baseline CRP level ≥3 mg/dL was a predictive factor for clinical remission after 8 weeks (OR: 4.77, *p* = 0.01) in a cohort of 134 UC patients treated with IFX [43]. In a prospective study on 21 patients with moderate-to-severe UC treated with GOL, non-responders had higher CRP baseline levels when compared with responders (8.0 vs. 4.7 mg/L, *p* = not significant) [35].

Similarly, baseline CRP levels were shown to be a predictive factor of response to IFX in a study of 226 CD patients where 76% of patients with CRP 45 mg/L had a response to IFX compared with 46% of normal CRP patients (*p* = 0.004) [44]. This correlation was in line with another trial where elevated pre-treatment CRP was associated with a better response than lower levels in CD patients treated with CER [45]. Moreover, among 718 CD patients treated with IFX, higher baseline CRP levels correlated with increased response when compared with normal levels [46]. Intriguingly, CRP levels were constantly higher in patients with LOR to IFX when compared with patients with sustained response (*p* = 0.001). In the first group, CRP levels remained significantly higher (median, 11.2 mg/L) and did not come back to baseline values (median, 18.2 mg/L; *p* = 0.039) [46]. Conversely, elevated baseline CRP levels were shown to be a predictor for dose escalation of ADA in a study on 720 CD patients (*p* < 0.05). Interestingly, altered baseline CRP levels were associated with failure of dose escalation (*p* = 0.02) [47].

Also, high pre-treatment level of Hb (≥11.5 g/dL) was a predictive factor for clinical remission after 8 weeks in a cohort of 134 UC patients treated with IFX (OR: 4.47, *p* = 0008) [43]. Similarly, Hb ≤9.4 g/dL correlated with primary non-response to IFX in a multicenter retrospective study on 191 UC patients (OR: 4.35) [48].

Finally, pre-treatment albumin level could also represent a valuable measure of anti-TNF-α therapy pharmacokinetic expectations in UC patients. Indeed, low serum albumin levels correlated with reduced IFX concentration and not a good response to IFX in a data analysis of 728 patients with UC [49]. In the above-mentioned prospective study on 21 UC patients treated with GOL, non-responders had lower baseline albumin levels when compared with responders (*p* = not significant) [35]. Probably, this could be related to a common clearance pathway for both albumin and anti-TNF-α antibodies.

#### 3.2.8. Immunologic Markers

Antibodies against microbial antigens could be useful for predicting response to anti-TNF-α. In UC, the absence of perinuclear antineutrophil cytoplasmic antibodies (p-ANCA) was correlated with better response to IFX in two studies on 90 and 100 UC patients, respectively [32,34]. Despite these data, serologic markers are not commonly used. Probably, they could have a broader application if used in combination with other predictive factors.

In CD, no relationship was found between the combination pANCA-positive/anti-saccharomyces cerevisiae antibody (ASCA)-negative and response rates to anti-TNF-α [50]. Conversely, speckled ANCA (sANCA) positivity was related to better response to IFX in a retrospective analysis of 59 CD patients [51]. However, a further study did not confirm this observation [16].

### 3.3. Treatment-Related Factors

Recent data showed that better response rates to anti-TNF-α could be achieved through the optimization of therapeutic regimen on the base of short-term clinical response, mucosal healing, drug trough levels and anti-drug antibodies.

#### 3.3.1. Short-Term Clinical Response

Recent studies evidenced that an early clinical response (i.e., within 3 months) to anti-TNF-α in UC is a positive predictor factor for long-term response. Different clinical and biological markers defined short-term response in these trials.

Absence of short-term clinical response was indicated as an independent predictor of colectomy (HR: 10.8, *p* < 0.001) in a single center study on 121 UC refractory patients treated with IFX [13]. These results were confirmed also on 134 UC patients treated with IFX, with response at week 2 indicated as a predictive factor for higher response rate (OR: 20.54, *p* = 0.006) [43]. In two retrospective studies on 30 and 48 UC patients, respectively, treated with ADA, the risk of surgery was significantly increased when clinical response was not achieved after 12 weeks of treatment [52,53]. Similar results were observed in a cohort of 23 UC patients treated with ADA [54]. Finally, achievement of early response at week 12 was a predictive factor of remission at week 54 in a cohort of 88 UC patients treated with ADA (OR: 4.17) [55].

In CD, data on early clinical response are confusing and no remarkable data are available. In this scenario, mucosal response is preferred as a predictive factor in the majority of trials.

#### 3.3.2. Mucosal Healing

Despite the absence of a clear agreement on its definition, mucosal healing is a definitive therapeutic target to achieve in the natural history of IBD, since it has been shown to be a positive prognostic factor for a sustained long term remission [56]. Commonly, mucosal healing is defined as the absence of mucosal ulceration in patients who showed these alterations at baseline endoscopic exam [56].

In ACT trials, early endoscopic amelioration at week 8 in moderate to severe UC patients treated with IFX was a predictive factor for improved clinical outcomes, lower colectomy risk at week 54 (*p* = 0.0004), increased rates of remission and steroid-free remission (*p* < 0.0001) [57]. Consistently, among 45 patients with acute, steroid-refractory UC treated with IFX, mucosal healing at 3 months significantly influenced long-term risk of colectomy (*p* = 0.02) [58]. Similarly, mucosal healing was the only factor influencing long-term response in a cohort of 134 UC patients treated with IFX (OR: 4.66, *p* = 0.04). 

In a cohort of 214 CD patients treated with IFX, mucosal healing correlated with an ameliorated long-term outcome mainly thanks to a reduced necessity of major abdominal surgeries (*p* < 0.0001) [59]. Similarly, complete mucosal healing in patients with early-stage CD and treated with IFX was correlated with significantly higher steroid-free remission rates after 4 years of therapy (*p* = 0.036) [60]. Retrospectively, mucosal healing assessed after 3 months of therapy with IFX was a predictor of ongoing response at 12 months in a cohort of 71 patients [61]. These data were confirmed in a prospective study on 42 CD patients treated for at least 3 months with ADA or IFX, where mucosal healing after 3 months of therapy was an important predictor for long-term endoscopic response (*p* = 0.01) [62].

#### 3.3.3. Trough Levels

The assessment of anti-TNF-α serum concentration has been directly correlated with better clinical and endoscopic outcomes and for this reason is becoming a commonly used analysis in daily clinical practice.

Casteele et al. conducted a retrospective study on 90 IBD patients (64 CD and 26 UC) treated with IFX and found that low IFX trough levels at week 6 and week 14 correlated with higher risk of antibodies to IFX (ATI) formation and IFX discontinuation. In particular, an IFX trough level at week 14 < 2.2 μg/mL was a predictive factor for IFX discontinuation due to persistent LOR or hypersensitivity reactions (74% specificity, 82% sensitivity, likelihood ratio 3.1; *p* = 0.0026) [63]. In a prospective study on 64 UC patients treated with either IFX or ADA, trough levels correlated with mucosa healing. Specifically, trough levels of anti-TNF-α necessary to achieve mucosal healing were 2.7 μg/mL for IFX and 10.3 μg/mL for ADA [64]. Furthermore, in a single center study on 73 UC patients treated with ADA, serum concentration of ADA ≥4.58 μg/mL at week 4 correlated with short-term response (OR 4.85; *p* = 0.009), while serum concentration of ADA ≥7 μg/mL at week 4 correlated with long-term response (OR 3.56; *p* = 0.025) [65]. In an observational study on 21 patients with moderate-to-severe UC treated with GOL, serum GOL levels were significantly increased in clinical responders compared with non-responders both at week 2 and 6 (10.0 vs. 7.4 μg/mL, *p* = 0.035 and 5.1 vs. 2.1 μg/mL, *p* = 0.037, respectively) [35]. Also, in the “Program of Ulcerative Colitis Research Studies Utilizing an Investigational Treatment” (PURSUIT) studies, clinical response correlated to GOL serum levels. In particular, the PURSUIT-SC study evaluated the efficacy of subcutaneous GOL induction regimen in anti-TNF-α-naïve patients with moderate-to-severe UC [66]. Instead, PURSUIT-M study evaluated GOL maintenance therapy in patients who responded to the GOL induction regimen [67]. In both trials, higher clinical response and remission rates correlated with higher GOL serum concentration. Specifically, serum levels of 2.5 μg/mL in the induction regimen and 1.4 μg/mL in the maintenance phase were shown to be the optimal target [68]. Interestingly, an ongoing prospective study, the “early MOnitoring of Response” (MORE), is tempting to establish the optimal GOL regimen by assessing the probability of clinical response at week 26 on the base of its pharmacokinetic data at week 6 [69].

According to an observational study on 483 CD patients treated with IFX, IFX trough levels >2.79 μg/mL were an independent predictor of remission (OR: 1.8; *p* < 0.001), defined as a C-reactive protein concentration of ≤5 mg/L [70]. Similarly, IFX trough levels >3 μg/mL were associated with a decreased risk of treatment failure (HR: 0.34) in a cohort of 84 CD patients [71]. Consistently, IFX trough levels ≥3 µg/mL during maintenance treatment was an important determinant of clinical and endoscopic CD outcomes in a post hoc analysis of 203 CD patients treated with IFX alone or in combination with Azathioprine (OR: 3.34) [7]. Moreover, among 168 CD patients treated with ADA, discontinuation due to LOR was directly related to low ADA trough serum concentration, which was evidenced more frequently in patients with antibodies against ADA [72]. In a cohort of 71 CD patients, high ADA trough levels were associated with disease remission (*p* < 0.001). In particular, a cut-off drug level 0f 5.85 μg/mL showed optimal sensitivity, specificity and positive likelihood ratio for remission prediction (68%, 70.6% and 2.3%, respectively) [73]. Similar results were found in another study of 40 CD patients treated with ADA. In this case, the median ADA trough levels were increased in clinical remission (6.02 μg/mL) compared with active disease (3.2 μg/mL; *p* = 0.012). Trough levels were also higher in those patients who achieved mucosal healing (6.5 μg/mL) vs. those who failed (4.2 μg/mL; *p* < 0.005). Interestingly, ADA trough levels <4.9 μg/mL were associated with the lack of mucosal healing (likelihood ratio, 4.3; sensitivity, 66%; specificity, 85%) [74].

Finally, also CER trough levels correlate with clinical and endoscopic response. Indeed, in a cohort of 89 CD patients, higher concentrations of CER at week 8 correlated with endoscopic response (*p* = 0.0016) and remission (*p* = 0.0302) at week 10. At week 54, CER trough levels correlated with endoscopic remission rates (*p* = 0.0206). Furthermore, a significant inverse relationship between CER trough levels and pre-treatment levels of C-reactive protein and body weight was described (*p* = 0.0014 and *p* = 0.0373, respectively).

Overall, these data evidence the importance of considering the pharmacokinetics of anti-TNF-α and the need for therapeutic drug monitoring with the end goal of treatment optimization.

#### 3.3.4. Antibodies to Infliximab (ATI)

Recent studies focused on the importance of anti-TNF-α monoclonal antibodies formation in influencing their therapeutic efficacy. This parameter should be considered in combination with anti-TNF-α serum concentration. Moreover, ATI are associated with increased risk of hypersensitivity [70].

In a cohort of 115 patients with UC treated with IFX, antibody-status seemed to influence neither clinical remission nor colectomy rates [75]. On the other side, in a retrospective study on 106 IBD patients, ATI was significantly higher in patients with LOR (median ATI 35 U/mL) compared to patients with maintained response. A cut-off value of ATI ≥10 U/mL yielding a sensitivity of 81% and specificity 90% was identified. Combined measurements of IFX trough levels and ATI showed higher accuracy for discriminating between clinical response types to IFX maintenance therapy [76]. These results were confirmed in another trial on 62 mixed IBD patients treated with IFX. Higher ATI correlated with LOR to IFX (*p* < 0.001) [77]. In the abovementioned study by Casteele et al., ATI were present in 53/90 patients, but then ATI disappeared in 15/53 patients (transient ATI). Importantly, the 68% of patients with stable high ATI levels discontinue IFX treatment vs. the 13% of patients with transient ATI (RR: 5.1; *p* = 0.0005). Thus, this study evidenced the possibility for ATI to be transient. In these cases, transient ATI are not always linked to a worse clinical outcome. On the other side, stable high levels of ATI are correlated with permanent LOR [63].

In the cohort of 483 CD patients treated with IFX, a cut-off ATI value <3.15 U/mL correlated with remission (OR: 0.57; *p* = 0.002), defined as a C-reactive protein concentration of ≤5 mg/L. Moreover, this observational study evidenced that the development of ATI could increase the risk of having an active disease also with low concentrations and in the presence of therapeutic drug levels during maintenance [70]. Finally, in a post-hoc analysis of “A Crohn’s disease Clinical trial Evaluating infliximab in a New long-term Treatment regimen in patients with fistulising Crohn’s disease” (ACCENT) I trial where 573 CD patients had been treated with different regimens of IFX, ATI were lower in patients treated with induction regimen followed by maintenance treatment compared to those treated with a single infusion. Overall, reduced ATI levels and increased clinical benefit were evidenced when an induction strategy was followed by maintenance therapy compared with a single dose and then episodic retreatment [78].

### 3.4. Gene Expression in Colorectal Mucosa

Although response to anti-TNF-α is likely to be multifactorial, genetic factors could be able to anticipate the personal response to anti-TNF-α. In fact, potential predictors for anti-TNF-α response have been considered by testing of targeted variants within candidate genes.

Toedter et al. conducted a study on 48 UC patients treated with IFX and analyzed gene expression after treatment. IFX had a significant modulatory effect on mRNA expression in responders. Genes affected were mainly related to inflammatory response, cell-mediated immune responses, and cell-to-cell signaling. On the other side, no modulation of T(H_1_), T(H_2_) and T(H_17_) was found in non-responders [79]. Similarly, gene-array analysis on colonic mucosal biopsies from UC patients before starting therapy with IFX showed a differential gene expression among responders and non-responders. In particular, the most important differences between the two groups were observed for osteoprotegerin, stanniocalcin-1, prostaglandin-endoperoxide synthase 2, interleukin-13 receptor α-2 and interleukin-11 [80].

Moreover, IFX was found to have opposite effects on Foxp3(+) Treg cells in blood and gut mucosa with specific results in responders. Indeed, IFX treatment was correlated to a significant and stable increase of CD4(+)CD25(+)Foxp3(+) Treg and of CD4(+)CD25(−)Foxp3(+) Treg cells in peripheral blood (both *p* < 0.0001), mainly in responders (both *p* < 0.05 compared to non-responders). A durable increase of circulating Foxp3(+) cells was associated with a sustained clinical response. Intriguingly, IFX therapy was able to down-regulate mucosal mRNA and protein expression of Foxp3 in UC and CD responders (both *p* < 0.001) but not in non-responders [81].

Another target has been clearly TNF-α. In a cohort of 59 UC patients with moderate to severe disease treated with IFX, baseline the unique independent predictive factor of clinical and endoscopic remission was TNF-α expression (*p* = 0.01 and *p* = 0.003, OR: 2.5 and 4.8, respectively) [82]. Among other cytokines, elevated baseline mucosal expression of IL-17 and IFN-γ was significantly linked to remission after induction therapy (OR: 5.4, *p* = 0.013 and OR: 5.5, *p* = 0.011, respectively) in a cohort of 74 UC patients treated with IFX [83].

The real impact of genetic polymorphisms to response to anti-TNF-α is still not well established. In a cohort of 90 UC patients, homozygosity for the high IBD risk IL-23R variants was found to be a predictive factor of response to IFX when compared to homozygosity for IBD-risk-decreasing IL23R variants (74.1% vs 34.6%; *p* = 0.001) [34]. 

In CD, the IBD5 gene (5q31) modulation was associated to non-response [84], while IgG Fc receptor IIIa (FC RIIIa) gene regulation was associated to response to IFX [85]. The latter has 2 variants (V and F), is expressed on macrophages and natural killer cells and is able to activate cytotoxic immune cells. Homozygous V/V patients showed 100% biologic response to IFX compared to 70% of the others [85].

TNF-α polymorphisms and response to anti-TNF-α agent have been evaluated in several studies with mixed results. CD patients homozygous for a TNF-α polymorphisms (LTA Ncol-TNFc-aa 3L-aa26 1-1-1-1 haplotype) were established to not respond to IFX [51], while a response to IFX was found in CD patients TNFR-1 + 36 [86]. However, these relationships were not confirmed in subsequent studies [85,87]. Although nucleotide-binding oligomerization domain-containing protein (*NOD*) 2 gene has been clearly associated to CD risk [1], no clear correlation has been evidenced between *NOD2* gene and response to anti-TNF-α [88,89].

Interestingly, since both ADA and IFX are able to induce cellular apoptosis [90], Hlavaty et al. developed and studied an apoptosis pharmacogenetic index (API) in a small retrospective study using 3 single nucleotide polymorphisms (SNPs): Fas ligand-843 C/T, Fas-670 G/A and Caspase9 93 C/T. They found that higher API score correlated with better response and remission rates to anti-TNF-α in both luminal and fistulizing disease [91].

## 4. Conclusions

Anti-TNF α agents are powerful and revolutionary means for the treatment of IBD, but primary non-response or loss of response occur in some patients. The right drug and the specific treatment strategy for each patient is the end goal for a personalized medicine. In this field, the identification of predictive factors for anti-TNF-α response in IBD patients is the first step to avoid unnecessary treatments and eventually to shift to other biologics (or small-molecule drugs) belonging to different pharmacological classes. However, in these years the majority of studies that have tried to investigate these factors have provided controversial results. Primarily, this could be due to different experimental settings and to the different definitions of disease remission used. Moreover, many studies included a small number of patients and most of them were retrospective. Consequently, short- and long-term outcomes of an anti-TNF-α treatment could substantially differ depending on the study, rendering data interpretation even more complex. This has produced so far a low use of predictive factors in daily clinical practice. Despite these objective difficulties, we have tried to summarize some predictive factors of efficacy of anti-TNF-α therapy with major evidences from literature. These factors are reported in Table 2 and Table 3, distinguishing between those that can be used in clinical practice from those not easily accessible.

Clearly, in the future, the creation of specific algorithms with the combination of multiple variables could deeply improve their predictive baseline strength before starting anti-TNF-α agents. At the same time, in the era of precision medicine, newly diagnosed IBD patients will need to have their genetic, microbiome and immune characteristics measured at time 0, then matched to the most appropriate biological or immunosuppressive treatment based on likelihood of response/adverse effects. This scenario obviously will not exclude the importance of clinician experience and intuition in practically applying this information to the specific patient.

## Figures and Tables

**Table 1:** Different parameters used for response assessment to anti-tumor necrosis factor (TNF)-α agents in ulcerative colitis and Crohn’s disease.

	Clinical Response	Endoscopic Response	Biologic Response
Ulcerative Colitis	UCDAI	Mayo endoscopic subscore	Hb levels
	Mayo score		CRP levels
	Colitis Activity Index		Calprotectin levels
	Colectomy Rate		
	Steroid-free remission		
Crohn’s Disease	CDAI	SES-CD	Hb levels
	Steroid-free remission	CDEIS	CRP levels
	Surgery rate		Calprotectin levels

UCDAI: Ulcerative Colitis Disease Activity Index; CDAI: Crohn’s Disease Activity Index; SES-CD: Simple Endoscopic Score for Crohn’s Disease; CDEIS: Crohn’s Disease Endoscopic Index of Severity; Hb: Hemoglobin; CRP: C-reactive protein.

**Table 2:** Predictive factors of response to anti-TNF-α available in clinical practice.

**Patient-related factors** Age (earlier age at the start of IFX was associated with better outcomes in patients with CD) Weight (weight below 82 kilograms was associated with increased rates of clinical remission in UC patients treated with ADA)
**Disease-related factors** ● Disease duration (shorter disease duration was associated with increased efficacy in CD patients) ● Disease severity (disease severity was associated with worse therapeutic outcomes in UC patients) ● Disease phenotype (inflammatory phenotype is a predictive factor of response to anti-TNF-α than a complicating phenotype) ● Laboratory (biological) factors ✓ High baseline CRP levels are predictive of response to anti-TNF-α in CD patients ✓ High baseline Hb levels are associated with better response in UC patients ✓ Low serum albumin levels are negatively correlated with response to anti-TNF-α in UC patients
**Treatment-related factors** Early clinical response ((i.d. within 3 months from starting therapy) is a predictive factor of long-term response in patients with UC) Mucosal healing (predictive factor for better therapeutic outcomes both in CD and UC patients) Trough levels of anti-TNF-α (anti-TNF-α serum concentration is directly correlated with better therapeutic outcomes in IBD patients) ATI (sustained high levels are associated to LOR)

IFX: infliximab; CD: Crohn’s disease; UC: ulcerative colitis; ADA: adalimumab; ATI: Antibodies to IFX; IBD: inflammatory bowel diseases; LOR: Loss of Response.

**Table 3:** Predictive factors of response to anti-TNF-α not yet available in clinical practice.

**Predictive factors** Increased CD4(+)CD25(+)Foxp3(+) Treg and CD4(+)CD25(−)Foxp3(+) Treg in peripheral blood are associated with response to IFX Elevated baseline mucosal levels of TNF-α are predictive of clinical and endoscopic remission in UC patients Elevated baseline mucosal levels of IL-17 and IFN-γ correlated with remission after induction therapy with IFX in UC Homozygous V/V variant of IgG Fc receptor IIIa (FC RIIIa) gene was associated with response to IFX in CD patients Higher API score correlated with better response and remission rates in CD patients

API: Apoptosis Pharmacogenomics Index.
